# Intersession reliability of population receptive field estimates

**DOI:** 10.1016/j.neuroimage.2016.09.013

**Published:** 2016-12

**Authors:** Jelle A. van Dijk, Benjamin de Haas, Christina Moutsiana, D. Samuel Schwarzkopf

**Affiliations:** a Experimental Psychology, University College London, 26 Bedford Way, London, UK; b UCL Institute of Cognitive Neuroscience, 17-19 Queen Square, London, UK; c Department of Psychology, University of Kingston, Kingston upon Thames, UK

**Keywords:** pRF, population receptive field, CMF, cortical magnification factor, fMRI, functional magnetic resonance imaging, BOLD, blood oxygenation level-dependent, HRF, hemodynamic response function., Population receptive fields, Intersession reliability, Retinotopic mapping, Natural images, Functional MRI

## Abstract

Population receptive field (pRF) analysis is a popular method to infer spatial selectivity of voxels in visual cortex. However, it remains largely untested how stable pRF estimates are over time. Here we measured the intersession reliability of pRF parameter estimates for the central visual field and near periphery, using a combined wedge and ring stimulus containing natural images. Sixteen healthy human participants completed two scanning sessions separated by 10–114 days. Individual participants showed very similar visual field maps for V1-V4 on both sessions. Intersession reliability for eccentricity and polar angle estimates was close to ceiling for most visual field maps (*r*>.8 for V1-3). PRF size and cortical magnification (CMF) estimates showed strong but lower overall intersession reliability (*r*≈.4–.6). Group level results for pRF size and CMF were highly similar between sessions. Additional control experiments confirmed that reliability does not depend on the carrier stimulus used and that reliability for pRF size and CMF is high for sessions acquired on the same day (*r*>.6). Ou*r* results demonstrate that pRF mapping is highly reliable across sessions.

## Highlights

• We assess the intersession reliability of population receptive field (pRF) estimates.
• Reliability of visual field position estimates in V1-3 is excellent (*r* >.8).
• Reliability of pRF size and local cortical magnification factor are good (*r*≈.3-.6), especially when tested on the same day (r>.6).
• Natural images are an effective stimulus for visual field mapping.

## 1 Introduction

Roughly 20% of the human cerebral cortex responds to visual stimuli and large parts of visual cortex are organized as visual field maps: neighboring points in the visual field are represented on neighboring parts of cortex (Saygin and Sereno, 2008, Sereno and Huang, 2006, Wandell et al., 2007). Since the discovery of one such visual field map in the primary visual cortex (V1) (Holmes, 1918, Inouye, 1909) many other retinotopic maps have been identified in higher visual areas such as V2, V3, V4, V3A, and V5/hMT (Wandell et al., 2007).

Functional magnetic resonance imaging (fMRI) can be used to map the retinotopic organization of the human brain non-invasively (Sereno et al., 1995). In recent years*, population receptive field* (pRF) *mapping* (Dumoulin and Wandell, 2008; see e.g. Williams et al. (2003) and Larsson and Heeger (2006), for earlier approaches) has gained popularity (see for instance Barton and Brewer (2011), Baseler et al. (2011), Dilks et al. (2009), Masuda et al. (2010), Masuda et al. (2008), Hoffmann et al. (2012), Hoffmann et al. (2003), Conner et al. (2007), Schwarzkopf et al. (2014)). In comparison to conventional phase encoded methods (e.g. Sereno, 1995), pRF mapping estimates both the location in the visual field to which a voxel is most responsive, and the *extent* of the area to which the voxel responds. Most commonly this entails fitting a forward model to a voxel's response time series (Dumoulin and Wandell, 2008, but see e.g. Lee et al. (2013), Greene et al. (2014), de Haas et al. (2014) for data-driven approaches). PRFs are often modelled as two-dimensional Gaussians with three parameters: the *x* and *y* position of the receptive field center in visual space, and its standard deviation σ, henceforth referred to as pRF size (Dumoulin and Wandell, 2008). Since the stimulus time course is known, the predicted neuronal response can be estimated by the overlap of the stimulus with the pRF at any given time. Assuming a linear relationship between neuronal activity and the elicited blood oxygenation level-dependent (BOLD) response (Hansen et al., 2004), the predicted BOLD response is generated by convolving the predicted neuronal time series with a hemodynamic response function (HRF). The pRF parameters of each voxel are fitted by minimizing the residuals between the predicted and observed BOLD time series.

Besides basic pRF center and size parameters, cortical magnification factor can be estimated. The CMF refers to the cortical distance between two points that represent a fixed distance in visual space (Daniel and Whitteridge, 1961). Local cortical magnification in V1 has been shown to correlate with individual visual acuity thresholds (Duncan and Boynton, 2003). More recently, the subjective strength of visual contextual illusions (Schwarzkopf and Rees, 2013; Schwarzkopf et al., 2011; Song et al., 2013), perceptual transition spread speed in binocular rivalry displays (Genç et al., 2013), visual search performance (Verghese et al., 2014), and even visual working memory capacity and the precision and strength of visual imagery (Bergmann et al., 2014, Bergmann et al., 2015) have been shown to correlate with the local surface area of early visual regions.

Crucially, however, in order to make claims about links between behavior and pRF properties, the reliability of pRF measures over time remains to be established. Thus far the only study related to this topic suggests high stability of pRF parameters between scanning sessions in a small sample (n=3) and across visual areas (Senden et al., 2014). Here we compare mapping reliability in a larger sample and for individual visual areas. Natural images have been shown to maximize blood BOLD responses in both early and higher visual areas (Rainer et al., 2001). Here we investigate the intersession reliability of pRF estimates obtained using natural images as mapping stimuli. Moreover, we additionally compare the intersession reliability of natural image mapping stimuli with more traditional mapping stimuli.

## 2 Materials and methods

### 2.1 Scanning sessions

To assess the reliability of pRF measures over time, we acquired two scanning sessions per participant. We used natural images as mapping stimuli for these sessions. To further compare natural images with more traditional mapping stimuli, we performed four additional scanning sessions for three of the original participants. These stimulus comparison scans were divided into two days of two sessions each. On one day, ‘dartboard’ stimuli (see below for description) were used for one mapping session, while ‘ripple’ stimuli (see below for description) were used for the other session. On the other day, natural images were used for both sessions. The first set of additional scans was acquired to allow for an assessment of the baseline reliability of the initial natural images scans compared to more traditional mapping stimuli. However, as there was a considerable time between the initial and additional scans, a direct comparison was undesirable. To this end, the set of additional natural image scans was acquired. This set also allowed us to calculate the maximum expected reliability for the natural image mapping stimuli. In the main study and the additional sessions, a ‘session’ refers to a set of MRI scans that ended with the participant being removed from the scanner.

### 2.2 Participants

Four authors (JAvD, BdH, CM, DSS) and twelve healthy adults (8 female, age range 19–36 years, 1 left-handed) with normal or corrected-to-normal visual acuity participated in the natural images reliability sessions. Participants completed two scanning sessions, with the second session taking place on average 48 days (range 10–114 days) after the first. Three authors (JAvD, BdH, DSS) further participated in the four additional stimulus comparison scans. Participants gave written informed consent and all experimental procedures were approved by the UCL Research Ethics Committee.

### 2.3 Stimuli

**Fig. 1:**
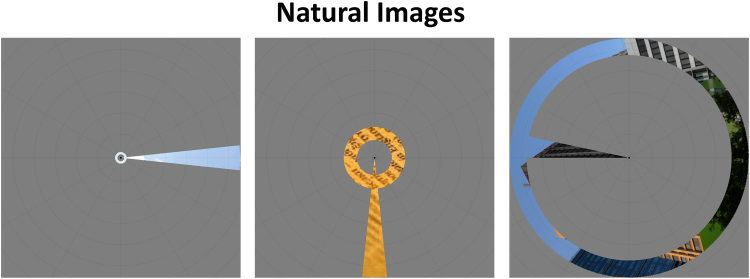
Example combined wedge and ring aperture mapping stimuli with natural images carrier patterns. From left to right, panels show the aperture rotating clockwise, illustrating presentations at approximately 90° intervals, omitting intermediate presentations. The ring aperture scales with eccentricity.

All stimuli were generated using MATLAB (Mathworks, 2012a) and the Psychophysics Toolbox (Brainard, 1997). For the natural image pRF mapping stimuli, 228 color images were extracted from Google Image searches. These images depicted cityscapes, mountains, beaches, forests, walls, textures, animals, faces, and pieces of writing in various scripts. Images were cropped and rescaled to 1080 × 1080 pixels. All parts of the image outside of 540 pixels from the center were set to medium grey, rendering the images circular. The order of images was randomized per run. A new image was displayed every 500 ms. All images were shown approximately twice per run. The images were overlaid with a uniform grey mask with a combination of a wedge- and ring-shaped aperture, so that the natural image was only visible within the boundaries of that aperture. The simultaneous wedge and ring mapping stimulus was selected because it has been found that this aperture produces subtly higher goodness-of-fit, as well as reducing acquisition time, compared to more traditional separate wedge and ring, and bar apertures. See Fig. 1 for mapping stimulus examples.

The wedge rotated around fixation in 60 discrete steps of 6° in clockwise or anticlockwise direction, stimulating different polar angle segments of the visual field in a systematic manner. Similarly, the ring stimulated different eccentricity bands in a systematic manner, either expanding or contracting. The direction of rotation and starting direction along the radial dimension were alternated between runs. Each step of the wedge and ring was presented for the duration of 1TR (1 s). The starting direction for the odd-numbered runs was clockwise rotating and expanding for the wedge and ring respectively. For the even-numbered runs, this was counter-clockwise and contracting respectively. The wedge stimulus completed 3 cycles per run, while the ring stimulus completed 5 cycles per run. The wedge stimulus was always presented from 0.07° from fixation to an eccentricity of 8.5° visual angle, with a polar angle size of 12° at all eccentricities. For the ring, the diameter of the outer boundary was determined by a logarithmic scaling functionf(x),

$$f\left(x\right)=17\;e^{(-4+\frac{1}{9}x)\;}(\mathrm{in degrees}\;\mathrm{visual angle})$$

with x increasing or decreasing per acquired volume in integer steps from 1 to 36 or vice versa. The inner boundary of the ring stimulus was at a minimum eccentricity of 0.25° and otherwise at 75% of the radius of the outer boundary. A fixation dot with a diameter of 0.13° was presented at all times at the center of the display. To further aid fixation compliance, low-contrast cross hairs covering the entire screen were present at all times. These cross hairs consisted of 12 radial lines, extending from right outside the fixation dot to the edges of the screen. These lines were equally spaced. Additionally, 11 concentric rings were presented, centered on fixation and increasing in radius with steps of 1.03° each. This pattern partly extended beyond the edges of the screen in the y direction. At the end of each scanning run there was a period of 45 s during which only the fixation dot and cross hairs were presented.

For each participant, and for each scanning session we also determined the individual's HRF in a separate scanning run. For this, we used sparse photic bursts. Each burst consisted of four consecutive 500 ms presentations of random images from the mapping stimuli set filling the entire circular mapped area. This was followed by 28 s of a blank screen in which only the fixation dot and crosshairs were presented. The run comprised ten such trials.

**Fig. 2:**
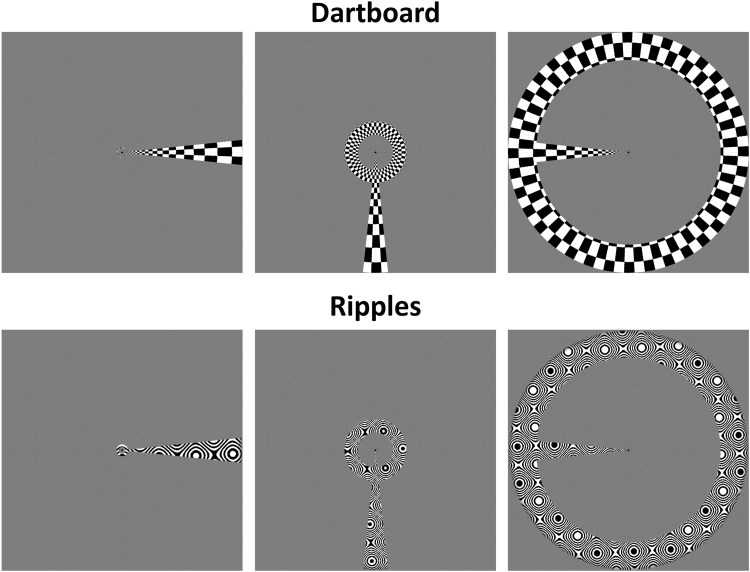
Example combined wedge and ring aperture mapping stimuli with dartboard and ripples carrier patterns. From left to right, panels show the aperture rotating clockwise, illustrating presentations at 90⁰ intervals, omitting intermediate presentations. The ring aperture scales with eccentricity.

Mapping stimuli for the stimulus comparison scans consisted of simultaneous ring and wedge apertures, containing either a ‘ripple’ pattern used in previous studies (Alvarez et al., 2015, Schwarzkopf et al., 2014) or a flickering ‘dartboard’ pattern (see Fig. 2 for examples of both stimuli). The ‘dartboard’ stimuli were divided into 72 polar checkerboard segments subtending 5° along the eccentricity axis. The width of segments was determined by taking the natural logarithm of the distance of each pixel from fixation (measured in pixels), multiplying it by 7, and rounding down. Odd and even numbered checkers were assigned minimum and maximum luminance and the checkerboard reversed contrast polarity at 2 Hz. All other parameters of the aperture and the timing were the same as for the natural images scans.

Stimuli were projected (resolution 1920×1080 pixels) on a screen (36.8×20.2 cm) at the back of the scanner bore and presented by means of a mirror on the head coil at a total viewing distance of 68 cm. While it is difficult to ensure exact viewing conditions across participants within a typical MRI setup, we took steps to minimize the errors in terms of the stimulated field of view. Participants were ‘landmarked’ consistently, based on the position of their eyebrows relative to the head coil. The mirror above their head was always placed at a fixed location on the coil. The position of the back-projection screen was fixed by means of markers and a plastic catch stuck to the top of the bore. We further ensured that the projected image on the screen had a consistent physical width. Stimuli therefore extended to an eccentricity of approximately 8.5° visual angle across participants and scanning sessions.

### 2.4 Procedure

For all mapping runs, participants were instructed to focus on the fixation dot at all times. During every 200 ms epoch, there was a probability of 0.03 that the dot temporarily changed color from black to one of 7 possible colors (white, red, green, blue, yellow, purple, and turquoise), with the restriction that any two event epochs must be separated by at least one non-event epoch. In order to ensure that participants remained attentive, participants were asked to monitor the color changes and indicate when the dot turned red by tapping their leg with a finger of their dominant hand. Additionally they had to indicate in the same way when the mapping stimulus showed a tartan pattern, which occurred approximately twice per run. No response data were collected. Eye movements were recorded using an eye tracker (Eyelink 1000) during the initial natural images sessions to assure that participants complied with the instructions and were attentive throughout the course of the experiment.

### 2.5 Scanning parameters

All imaging data were acquired on a Siemens Avanto 1.5T scanner with a 32-channel Siemens head coil with the front part removed (to avoid restrictions of the field of view), leaving 20 effective channels. Functional T2*- weighted multiband 2D echo-planar images were acquired using a CAIPIRINHA sequence (Breuer et al., 2005) with a voxel size of 2.3 mm isotropic, 36 slices, field of view (FOV)=96×96 voxels, repetition time (TR)=1 s, echo time (TE)=55 ms, flip angle=75°, acceleration factor=4. The collected slice package consisted of transverse slices. This package was tilted so that it was parallel to the calcarine sulcus for each subject, and positioned such that the central slice was aligned with the calcarine. Each participant completed 6 mapping runs per scanning session. 235 volumes were collected per run. The first ten volumes for each run were removed to allow the signal to reach equilibrium. The HRF acquisition scan lasted 310 TRs (TR=1 s) in total. In each session we also collected a 10 min run of resting state data. The screen was turned off and participants were instructed to close their eyes but to remain awake. In one session this run comprised 610 TRs of the same pulse sequence, while in the other session the run comprised 203 TRs of a conventional (non-multiband) sequence (TR=3 s). Whether this conventional sequence was used in the first or second session was counterbalanced across participants. These resting state data were not analyzed further in this study. Finally, a T1-weighted anatomical magnetization-prepared rapid acquisition with gradient echo (MPRAGE) scan was acquired at a resolution of 1 mm isotropic (TR=2730 ms, TE=3.57 ms) during one of the natural image mapping sessions.

### 2.6 Data pre-processing

Functional images were mean bias corrected, realigned and unwrapped, and co-registered to the structural scan (Friston et al., 1995a, Ashburner and Friston, 1997, Ashburner and Friston, 2005, Andersson et al., 2001, Hutton et al., 2002) using SPM8 (http://www.fil.ion.ucl.ac.uk/spm). All further analyses were performed using custom MATLAB code. The time series for each voxel in each run was linearly detrended, z-score normalized and averaged across runs, separately for both stimulus directions. For delineation of the regions of interest only, the averages for the two scanning sessions were further averaged *across* sessions. The resulting averages for the two stimulus directions were then concatenated. Next, the data were projected onto a 3D reconstruction of the grey-white matter boundary obtained using FreeSurfer (Dale et al., 1999, Fischl et al., 1999). For each vertex we used the affine transformation from the structural scan to the co-registered functional and determined the functional voxel that fell at the median position between the grey-white matter surface and the pial surface. Only vertices in the occipital lobe were included for further analyses.

### 2.7 Parameter estimation

For the main analysis, a canonical HRF was used for parameter estimation. The canonical HRF used was an average of individual HRFs (*n*=26) from de Haas et al. (2014) (peak latency=5.53 s, undershoot latency=16.9 s, ratio of response to undershoot=1.02, amplitude=1.47). The same HRF was used for parameter estimation for the stimulus comparison scans. Additional analyses using individual HRFs yielded virtually identical results (see below and Supplementary Fig. S2; c.f. Dumoulin and Wandell, 2008).

The forward modeling approach used was similar to the one in Dumoulin and Wandell (2008). PRFs were modeled as two-dimensional Gaussians g(x,y),

$$g\left(x,y\right)=e^{-\left(\frac{{\left(x-x_{0}\right)}^{2}+{\left(y-y_{0}\right)}^{2}}{2\sigma^{2}}\right)}$$

where $(x_{0},y_{0})$ is the center and σ is pRF size, all in degrees visual angle. For each vertex (cortical location), center and size parameters were estimated in a two-stage process. First, an exhaustive grid search approach was used for coarse parameter estimation. Spatial smoothing (Gaussian smoothing kernel, full width at half maximum (FWHM)=5 mm) was applied to an inflated spherical model of the cortical surface. Subsequently, a search space was generated (15×15×34) containing plausible values for the pRF center and size parameters. Next, a time series was generated by calculating the overlap between the stimulus time series and the candidate pRF parameters, and subsequently convolving this with the canonical HRF to get a set of predicted time series. Highly similar results were obtained when using the subject-specific HRF for this step (see Supplementary materials). The best fitting prediction in the search space was estimated for each vertex by calculating the Pearson correlation between the observed fMRI data and the predicted time series. For the second stage of parameter estimation, the estimates from this coarse fit were used as initial values in an optimization procedure. For this we used unsmoothed data. Optimal parameters were determined by minimizing the sum of squared errors between the predicted and observed time series. For this, a simplex-based optimization method was used (Lagarias et al., 1998, Nelder and Mead, 1965). In addition to the three pRF parameters, this fine fitting phase also included a scaling parameter estimating the overall response strength (β). We applied a modest smoothing kernel of FWHM=3 mm to the estimated parameter maps on the spherical surface in order to calculate the local CMF . Unsmoothed data were used for all other pRF parameters. Intersession reliability results were very similar when using smoothed pRF parameters or a subject-specific HRF instead (See Supplementary Figs. S1 respectively S2).

### 2.8 Definition of visual areas

PRF center parameters were transformed into polar angle and eccentricity, color-coded, and displayed as projections on the inflated cortical surface of individual hemispheres in Freesurfer (Dale et al., 1999, Fischl et al., 1999). We delineated the boundaries of retinotopic regions manually along mirror reversals in the polar maps, assisted by eccentricity maps for identification of foveal representations. Retinotopic regions of interest that were delineated included V1, V2, V3, V3A, and V4. Higher extrastriate regions were not included as they could not be readily delineated in every participant. Boundary definitions were based on Wandell and Dumoulin (2007). The inner boundary of V3A was defined by a lower vertical meridian reversal perpendicular to the superior boundary of the dorsal part of V3. The outer boundaries of the regions of interest were defined by the outer boundaries of meaningful visual responses, i.e. where expected polar angle representations for a given region of interest were visible. Only vertices with a model fit of R^2^ > 0.1 and a center position between 0.5–7.5° visual angle were included in the analysis.

### 2.9 Reliability estimation

To assess the reliability of pRF measures, estimates of pRF size, eccentricity, polar angle, and CMF were correlated between sessions on a vertex-by-vertex basis for each hemisphere. This was done for each region of interest separately. The vertices falling within the boundaries of these regions were defined by the labels of the delineated regions of interest. Labels based on data from the combined first and second scanning session were used to define regions of interest; however, since the maps were virtually identical for the two scanning sessions, using labels from either session resulted in extremely similar results (data not shown). Reliability measures were carried out on unsmoothed parameter maps from the fine-fitting procedure for eccentricity, polar angle, and pRF size. For CMF, surface-smoothed (Gaussian kernel with FWHM=3 mm) maps were used because the calculation of CMF assumes a smooth gradient of visual field representations. To compare parameter estimates between sessions, Spearman's rank correlation coefficient (Spearman's rho) was calculated for pRF size, eccentricity, and CMF for each hemisphere and region of interest. Polar angle estimate correlations for each vertex were calculated using the circular correlation coefficient $\rho_{\mathrm{cc}}$ (Jammalamadaka and Sengupta, 2001, p. 176).

As correlation sampling distributions are not normally distributed, all correlation coefficients were converted to standardized-scores using Fisher's z transformation (Fisher, 1915). Subsequently, z-scores were averaged per parameter and region of interest, and this average was transformed back into r-scores, resulting in the average correlation between sessions across participants for all parameters and regions of interest separately. The same procedure was followed to assess the intersession reliability between sessions using other mapping stimuli. For natural images runs, a repeated measures analysis of variance (RM-ANOVA) was performed on the first level z-transformed data using SPSS21 (IBM, 2012), with parameter (eccentricity, polar angle, pRF size, and CMF) and region of interest as within-subject factors.

To further investigate the reliability of pRF parameters obtained using natural images, unsmoothed maps were used for pRF size, and smoothed for CMF. Within each individual hemisphere and for a given region of interest, we calculated the mean pRF size and median CMF for an eccentricity band 1.5° in width. We used a sliding window approach in which we smoothly increased the eccentricity of this band in 100 steps and calculated the summary statistic across the band at each eccentricity. Then, we calculated the group mean and standard error of the mean for every eccentricity across participants and all hemispheres for each region of interest and parameter. Only a qualitative comparison was made for these group plots (cf. Dumoulin and Wandell, 2008; Harvey and Dumoulin, 2011). To further estimate the intersession reliability of these data we then calculated the Spearman correlation across participants separately for each eccentricity band. To limit the skew introduced by low quality data, we only included eccentricities for which there were at least data from 6 participants in both sessions.

### 2.10 Eye movements and head motion

Eye movements were recorded at 60 Hz. For each participant we calculated the proportion of valid samples, and as an (inverse) indicator of fixation stability the average median absolute deviation (MAD) of gaze across epochs for both the horizontal and vertical axes. We tested a relationship between (lack of) fixation stability and pRF reliability. For this we calculated Spearman's rho between the indicators of gaze stability on the one hand and the mean intersession reliability across visual regions of interest on the other.

Six rigid body head motion parameters were estimated for each run as part of the image realignment in SPM8 (http://www.fil.ion.ucl.ac.uk/spm/). We calculated the mean translational motion per participant by calculating the mean variance of translational regressor estimates across runs and axes. Additionally, we calculated the translational motion and then averaged that across runs and axes for each participant. The same indicators were calculated for mean rotational motion. To test a relationship between pRF reliability and head motion we correlated these motion indicators with the mean intersession reliability across visual regions of interest using Spearman's rho.

## 3 Results

### 3.1 Visual field maps

**Fig. 3:**
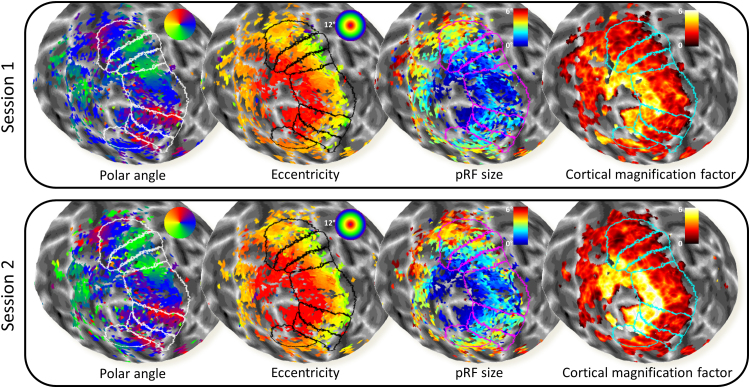
Example visual field maps for both sessions for the same (left) hemisphere of one participant. From left to right, polar angle, eccentricity, pRF size, and cortical magnification factor estimates are displayed. Enclosed areas denote delineated visual areas. Dorsal and ventral portions were pooled for all analyses of both V2 and V3. Units for CMF are mm/degree visual angle. R^2^ threshold for all maps is 0.05. CMF data were based on smoothed visual field maps but all other maps are unsmoothed parameter maps.

Fig. 3 shows an example of visual field maps for one hemisphere for both session. Polar angle, eccentricity, pRF size, and CMF estimates are displayed.

### 3.2 Parameter estimate reliability

**Fig. 4:**
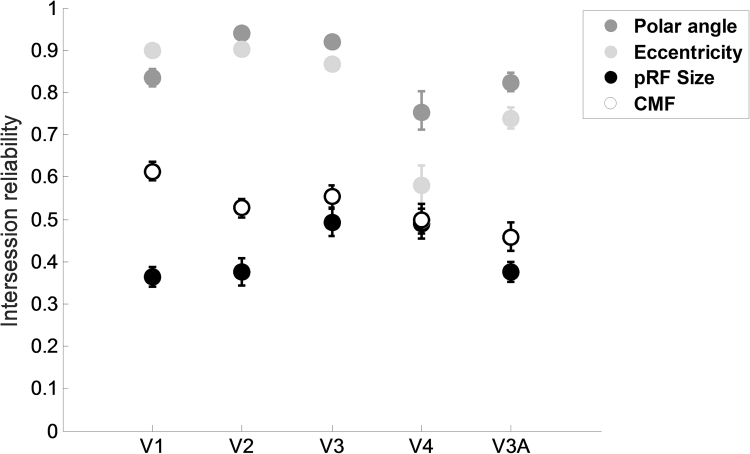
Intersession reliability estimates for polar angle, eccentricity, pRF size, and CMF for all regions of interest. Error bars denote ±1 standard error of the mean. If there are no error bars visible, they are smaller than the symbol. For eccentricity, pRF size, and CMF, mean Spearman's rho is displayed, while the mean circular correlation is displayed for polar angle.

See Fig. 4 for the average intersession reliability for all estimated parameters per region of interest, and Table 1 for a summary table. Polar angle estimate reliability was high for all regions of interest (minimum *r*=.75 (V4)). The same held for eccentricity estimate reliability (minimum *r*=.58 (V4)). CMF and pRF size reliabilities were generally lower (minimum *r*=.46 (V3A) for CMF, and minimum *r*=.36 (V1) for pRF size). Overall, parameter reliability was higher for early visual areas than for V3A and V4 (with the exception of pRF size). Parameter estimate reliability using smoothed data or individual HRFs revealed no large differences in the pattern of results (see Supplementary Figs. S1 and S2 respectively). This is in line with Dumoulin and Wandell (2008), who showed that the shape of the HRF only has a small effect on pRF estimates. Fixation compliance was also very stable for both scans (see Supplementary Fig. S3).

A repeated measures analysis of variance revealed significant main effects of parameter (*F*(1.6,24.2)=294.6, *p*<0.001) and region of interest (F(4,60)=39.8, p<0.001) on reliability estimates. There also was a significant interaction between parameter and region of interest (F(4.1,62.0)=22.7, p<0.001, Greenhouse-Geisser corrected). Subsequent pairwise comparisons showed that polar angle reliability was significantly higher than for all other estimates (Mean Difference=0.2*z* (Eccentricity)*,* 0.7*z* (CMF)*,* and 0.9*z* (pRF size), *p*<.05, *p*<0.001, *p*<0.001 *respectively*). Eccentricity reliability was significantly higher than both pRF size and CMF reliability (Mean Difference=0.7, 0*.*6*z,* both *p*<0.001). CMF reliability was significantly higher than pRF size reliability (Mean Difference=0.1*z, p*<0.001).

### 3.3 CMF and pRF size relationship with eccentricity

**Fig. 5:**
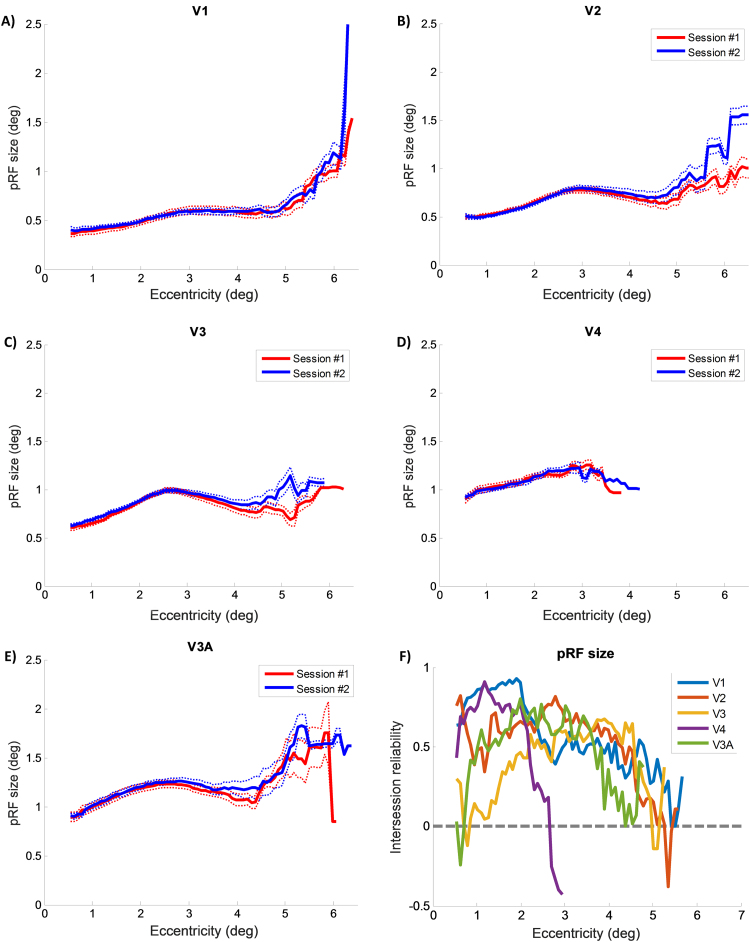
A-E) Average estimated pRF size for each eccentricity and visual area for both natural images sessions. Red: first session. Blue: second session. Thick lines indicate the group mean for each eccentricity. Dotted lines denote ±1 standard error of the mean (SEM). Note the high degree of similarity between sessions for both the group means and fitted curves. F) Intersession reliability for each eccentricity. Note that lower reliability is to be expected for bin-wise analyses because of the restricted variance and low number of vertices per bin.

Fig. 5 shows the group average of pRF size plotted against eccentricity for all areas, for both sessions. As expected, pRF size generally increased with eccentricity. Moreover, average pRF sizes increased across areas along the visual hierarchy. For V1 and V2, the fitted functions and average pRF sizes were highly similar between sessions for all eccentricity bins up to around 5°. Beyond that, edge artifacts began to limit the reliability of these results and data above threshold could only be obtained in a subset of participants. Average pRF size in V3 and V3A were also highly similar between sessions at low eccentricities, but deviated at eccentricities above 4°. In V4, average pRF size was very similar across the range but since the representation in V4 is very biased towards the central visual field, data were only obtained up to an eccentricity of 4°. Fig. 5F shows the intersession reliability (Spearman's correlation) plotted against eccentricity for each visual area. This revealed that reliability was generally strong (0.5–0.9) up to an eccentricity of 4–5° but beyond this it fell off sharply. In V4, where data above threshold were only obtained for the most central visual field, reliability already dropped off sharply beyond 2°. Moreover, in V3 and V3A reliability in the very central eccentricities (0.5–1.5°) were also poor but rose to the level of other areas within the range of 1.5–4°.

**Fig. 6:**
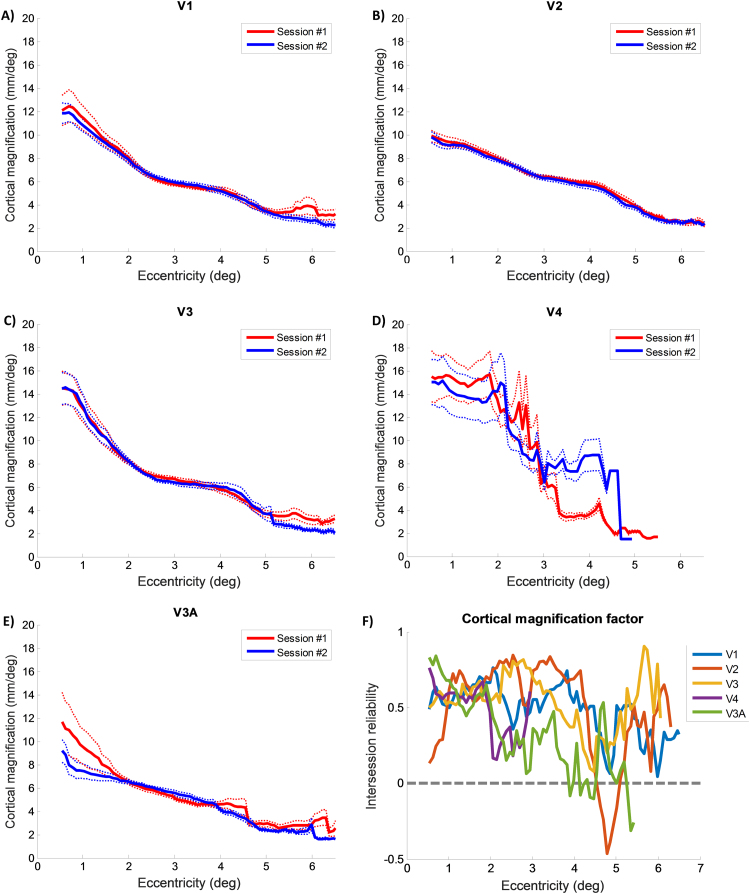
A-E) Average estimated CMF for each eccentricity and visual area for both natural images sessions. Red: first session. Blue: second session. Thick lines indicate the group mean for each eccentricity. Dotted lines denote ±1 standard error of the mean (SEM). Note the high degree of similarity between sessions for both the group means and fitted curves. F) Intersession reliability for each eccentricity. Note that lower reliability is to be expected for bin-wise analyses because of the restricted variance and low number of vertices per bin.

Fig. 6 shows the group average CMF plotted against eccentricity for all areas for both sessions. In line with expectations, CMF estimates decreased with eccentricity. Group averages of CMF were all highly similar between scans. We observed the largest deviations between sessions in V3A and particularly in V4. Fig. 6F shows the intersession reliability against eccentricity. Reliability in all areas except V2 was high (0.5–0.8) for the very central eccentricities (1–2°). The reliability of the higher areas dropped somewhat in the intermediate eccentricity range (2–4°). Beyond 4° the reliability of all visual areas then fell off although reliability in V1-V3 generally remained above zero.

### 3.4 Intersession reliability of alternative mapping stimuli

**Fig. 7:**
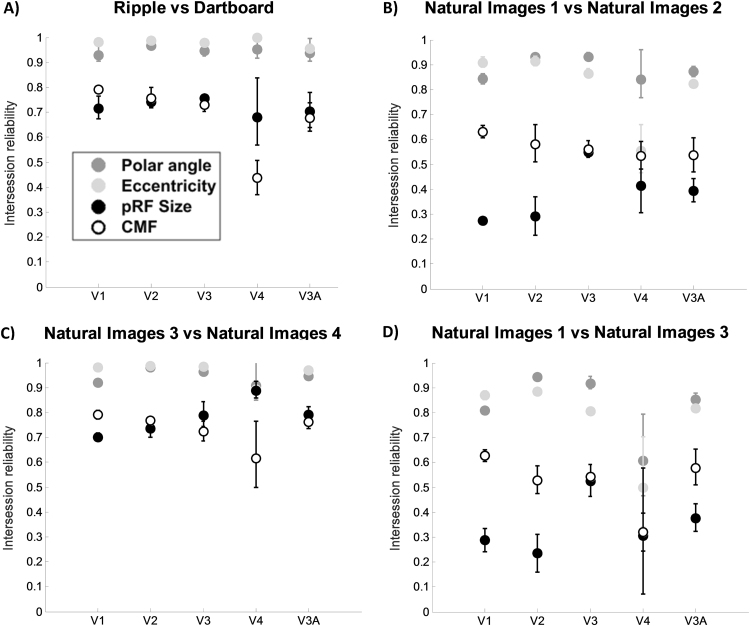
Intersession reliability estimates for different mapping stimuli/sessions (n=3). Error bars denote ±1 standard error of the mean. If there are no error bars visible, they are smaller than the symbol. Symbols denote average intersession reliability for polar angle (dark gray), eccentricity (light gray), pRF size (black) and CMF (white) estimates. For eccentricity, pRF size, and CMF, mean Spearman's rho is displayed, while the mean circular correlation is displayed for polar angle. Comparison of: A) ripple and dartboard stimuli, acquired on the same day, B) initial natural image sessions, acquired 38, 39, and 48 days apart, C) two natural image sessions, acquired on the same day, D) first initial natural image session with the third natural image session.

Within a small subset of three participants we acquired additional mapping data using ripple and more traditional dartboard stimuli (in separate sessions). The correlation between maps acquired with these stimuli showed markedly greater reliability across all measures (Fig. 7A) compared to the reliability of the original natural images sessions for the same three participants (Fig. 7B). This drop in intersession reliability for natural images is particularly pronounced for pRF size and CMF (a drop of ≈0.3 for pRF size and CMF while reliability polar angle and eccentricity drops only by ≈0.1). However, the former were acquired on the same day (albeit in separate sessions), while the sessions using natural image stimuli were separated in time (range: 10–114 days). We therefore ran another two natural images sessions acquired on the same day (Fig. 7C). This allowed us to test the same-day intersession reliability for natural image stimuli. This reliability was even subtly greater than that between ripple and dartboard scans, with nearly perfect reliability for polar angle and eccentricity estimates and pRF size and CMF estimates showing a re-scan reliability of ≈0.7–0.8. Intersession reliability comparing the first natural images mapping session to the traditional third natural image session, show a similar pattern as the initial natural images reliability estimates (Fig. 7D). Similar reliability results were found for any other pair of sessions that were not acquired on the same day (Fig. S4).

### 3.5 Eye movements and head motion

Eye movement data were collected for 27 out of 32 sessions. In those, an average of 88.7% (*SEM*=3.4%*, range* 20.6–99.7%) of samples were valid. Mean median absolute deviation (MAD) across epochs was 0.61° visual angle (*SEM*=0.09). The correlations between average gaze MAD and mean intersession reliability estimates for pRF size, CMF, polar angle, and eccentricity, were *ρ*=−.53*, p*=.04; *ρ*=−.17*, p*=.53; *ρ*=−.47*, p*=.07; and *ρ=*.03*, p* =.93 respectively. Especially the CMF correlations appeared to be driven by one participant who showed the largest gaze MAD (see Supplementary Fig. S4). Excluding this participant from the eye movement analysis resulted in gaze MAD correlations of *ρ*=−.43, *p*=.11; *ρ*=.01, *p*=.98; *ρ*=−.37, *p*=.18; and *ρ*=.17, *p*=.54 with pRF size, CMF, polar angle, and eccentricity respectively. The correlations between average translational motion and mean intersession reliability estimates for pRF size, CMF, polar angle, and eccentricity, were *ρ*=−.43, *p*=.10; *ρ*=−.25, *p*=.34; *ρ*=−.44, *p*=.09; and *ρ*=−.09, *p*=.73 respectively. The correlations between average rotational motion and mean intersession reliability estimates for pRF size, CMF, polar angle, and eccentricity, were *ρ*=.15, *p=*.57; *ρ*=.32*, p*=.23; *ρ*=.10, *p*=.71; and *ρ*=.09, *p*=.75 respectively.

## 4 Discussion

Our findings provide evidence for the reliability of visual field mapping using pRF modeling (Dumoulin and Wandell, 2008, Schwarzkopf et al., 2014). Moreover, we show that natural images with a combined wedge and ring aperture are well suited as mapping stimuli compared to more traditional stimuli. Our results suggest that the intersession reliability of eccentricity and polar angle estimates is very high, consistent with the assumption that the overall architecture of visual field maps is stable over time. In contrast, estimates for pRF size and cortical magnification factor show a larger variability between sessions but are nonetheless strongly correlated (*r*=.36–.49 and *r*=.46–.61 for pRF size and CMF, respectively). V1-3 reliability estimates are overall highest compared to other visual areas of interest. Group averages for pRF size and CMF for different eccentricities show a high degree of similarity between sessions, supporting the reliability of visual field estimates over time. The intersession reliability is particularly strong for the central part of the visual field but decreases fairly sharply towards the outer range of the mapped field beyond 5°. This suggests artifacts at the edge of the stimulated region (at 8.5°) affect the reliability of pRF parameters. The pRF sizes we observed are highly consistent with previously reported values up to an eccentricity of approximately 5° for V1 and V2 and 3° for V3 (Fig. 7 in Alvarez et al. (2015); Fig. 9 in Dumoulin and Wandell (2008); and Fig. 4A in Harvey and Dumoulin (2011). At higher eccentricities, the variance of our estimates increased and values deviated from previous reports (higher in V1 and V2, lower in V3).

Regardless of the carrier stimulus used, reliability for pRF size and CMF is considerably greater for sessions acquired on the same day compared to those separated in time. The intersession reliability for the dartboard and ripple stimuli is very high for all visual areas and parameter estimates. Similarly, the intersession reliability for two separate natural images stimuli sessions acquired on the same day was very similar to the results from the dartboard and ripple intersession reliability. Comparing the first natural image session with third natural image session (Fig. 7D) or any other sessions not acquired on the same day (Fig. S4) gave reliability results in line with those for the initial natural images mapping sessions. Taken together, this suggests that the factor limiting reliability is whether or not sessions were acquired on the same day, rather than the stimuli used for mapping.

The drop in pRF size and CMF reliability for separated sessions might in part be explained by the second-order nature of these parameters. There is a modest drop in reliability for polar angle and eccentricity estimates as well, which might have an exacerbated effect on the size and CMF parameters, which depend on accurate estimates of visual field position. Another potential reason for this is that differences in head position are probably greater for scans taking place on different days. For instance, the memory foam cushions used to pad in the participant's head would have remained the same between the two sessions on the same day whereas this was not necessarily the case for sessions acquired many days apart. Also, the scanner operator would always have been the same for sessions acquired on the same day whereas for sessions conducted on different days this was not always the case. So, for scans on different days small positional differences might well be greater – despite the fact that we standardized the positioning of participants’ eyes along the z-axis with fixed coil markings to keep the viewing distance constant. Additionally, head orientation relative to the head coil is likely to affect the recorded signal: the highly folded cortex gives rise to a wide distribution of angles between the cortical surface and B_0_ (Cohen-Adad et al., 2012). Recent evidence suggests that the local folding of the cortex gives rise to variations in the BOLD-signal of as much as 40% of the total signal (Gagnon et al., 2015). Differences in head orientation affect the distribution of angles of the cortical surface. Moreover, the absolute distance between the cortical surface and channels of the head coil is very small to begin with, so small differences in head positioning (e.g. small rotational differences) can have big effects on this distance and its relative distribution across the cortical surface. Therefore, slight variations in head orientation *between* scanning sessions might result in a relatively large differences in recorded BOLD-responses, ultimately contributing to the lower observed reliability between sessions acquired on different days. The head motion *during* a session, given it is not too high (smaller than one voxel on average), does not have a large effect on intersession reliability (Section 5.5).

The time between scans may also be a determining factor for reliability in and of itself. While regular quality assurance procedures are usually in place, we cannot rule out additional fluctuations in the image acquisition. Biological factors such as the general arousal and physiological state of the participant might also have differed between sessions, which could have additionally affected the reliability. These factors would likely be relatively similar for sessions acquired on the same day but unlikely to be so for sessions conducted on different days. For instance, while most scans would take place during normal office hours, for practical reasons we could not ensure that scans would be conducted at a similar time of the day for each participant.

The accuracy of pRF size estimates can also be influenced by a number of physical factors, such as eye-movements. These factors could have potentially increased variability in the pRF size estimates but are unlikely to consistently bias eccentricity and polar angle estimates. The latter claim is supported by the eye movement data, which show that the reliability of eccentricity and polar angle estimates were high, even for participants with reduced gaze stability. It has been shown that in order to bias pRF size estimates substantially, the extend of eye movements has to be large (Levin et al., 2010), which is in line with our findings that pRF size and CMF estimates appeared to be only slightly reduced for a single participant with the lowest gaze stability.

CMF is defined as the cortical distance between two points that represent locations in the visual field separated by a fixed distance in visual space. The generally lower reliability for CMF estimates, compared to eccentricity and polar angle estimates, could be caused by a number of factors. First of all, CMF is a second order estimate, as it is derived directly from eccentricity and polar angle estimates (see above). Therefore, the variance for CMF estimates is likely to be larger than that for polar angle and eccentricity, as observed in the current data. This then results in a lower CMF estimate reliability between sessions. Secondly, as CMF is a ratio measure, it is exponential and more sensitive to small intersession differences than the other parameters.

An important source of data noise stems from the fact that the collected data are based on a hemodynamic measure. These measures are only indirectly related to neuronal events (Logothetis, 2008), thus creating additional measurement error. A strong influence on the accuracy of hemodynamic response measurements is the signal-to-noise ratio (SNR) of the scanner. At a now conventional magnetic field strength of 3 T, the typical SNR is around 50 for voxels of ≈10 mm^3^ (Murphy et al., 2007). This requires about 860 time points to guarantee detection of a signal change of 1% (Glover, 1999). As BOLD changes are typically less than 1%, an even larger number of acquisitions is desirable for reliable signal detection. The used scanner had a magnetic field strength of 1.5 T and thus a somewhat lower SNR, although this is at least in part compensated for by the use of a state-of-the-art 32-channel head coil and the high-SNR pulse sequence we used. Our earlier results using a 3 T scanner, show a clear presence of LO and MT+. In our current data, these visual areas were not clearly distinguishable, which might be due to the difference in SNR between 1.5 T and 3 T scanners or the fact that we only used 20 effective channels covering the posterior part of the brain. The present findings likely provide a lower bound to the reliability that can be expected from experiments using pRF modeling when using scanners with stronger magnetic fields.

The combined wedge and ring aperture design of the used stimulus has an inherently asynchronous cycle speed for the polar angle and eccentricity dimensions. However, in a comparison with more traditional, separate wedge and ring aperture runs, the combined aperture show no appreciable differences in the parameter estimates. Nevertheless, a stimulus design with an identical cycling speed for both polar angle and eccentricity (in separate sessions) or a sweeping bar design may be more appropriate for some applications.

The observed - somewhat peaked - nature of the pRF versus eccentricity curves in Fig. 5, is a feature previously reported in pRF data (see for instance Fig. 8 in Dumoulin and Wandell (2008); and Fig. 1 in Harvey and Dumoulin (2011), as well as Fig. 1 in de Haas et al. (2014) for less extreme examples). This may be an artifact of the greater variability of pRF size estimates at more peripheral eccentricities, and general miss-estimation of pRFs that extend beyond the outer edge of the mapping stimulus. One indication that this may have played a role here is the relatively large between-subject variance (as reflected by the larger error bars) in our data for the outer eccentricity bins. However, as our group averages show, this feature was very consistent across our two scanning sessions. Further research is required to understand the source of this feature conclusively.

In this study we modelled pRFs as simple 2D-Gaussians. However, there is evidence from single cell studies that center-surround suppression plays a role in retinotopic maps on the neuronal level (Hubel and Wiesel, 1965), with suppressive strength and spatial extent increasing in higher visual areas (Kastner et al., 2001). Although the link between surround-suppression on the neuronal level and the voxel level is still unclear, it has been shown that incorporating surround-suppression in the pRF model results in a better data fit (see e.g. Kay et al. (2013), Williams et al. (2003), Zenger-Landolt and Heeger (2003), Zuiderbaan et al. (2012)). These center-surround pRFs can be modelled as the difference of two Gaussians (DoG). Thus implementing a DoG pRF model could potentially improve mapping reliability, although at the same time the need to fit two additional free parameters may *reduce* reliability.

Here we provide evidence that visual field maps in healthy adults are generally stable over temporal intervals spanning up to several months. However, the properties of retinotopic maps might change during development or as a consequence of various pathologies, e.g. (Conner et al., 2007, Hoffmann et al., 2012). Moreover, little is known about whether retinotopic maps are hereditary or shaped by environmental factors. The ability to obtain highly reliable estimates of retinotopic tuning in the first place – which we document here – is a requirement for addressing these questions.

In conclusion, we show that eccentricity and polar angle estimates derived by pRF mapping are very stable over time, while CMF and pRF size estimates are very reliable across scanning sessions on a single day, but less so for scans separated by longer intervals. Additionally, we provide evidence that natural images are effective mapping stimuli and provide very similar estimates to more traditional mapping stimuli. These findings provide an important basis for linking pRF estimates to behavior or comparing such estimates across different populations.

## Data availability

The processed pRF data and the code to reproduce the analysis of the main results (comparing two sessions using natural images as stimuli) are available for download at the following link, while all other anonymized data are available upon request.

http://dx.doi.org/10.5281/zenodo.54710

## Figures and Tables

**Table 1:** Summary of intersession reliability estimates for polar angle, eccentricity, pRF size, and CMF (cortical magnification factor). Numbers denote mean reliability for each parameter and visual region of interest; numbers between brackets denote SEM (standard error of the mean). For eccentricity, pRF size, and CMF, mean Spearman's rho is displayed, while the mean circular correlation is displayed for polar angle.

	**V1**, *M (SEM)*	**V2**	**V3**	**V4**	**V3A**
**Polar Angle**	.83 (.02)	.94 (.01)	.92 (.01)	.75 (.05)	.82 (.02)
**Eccentricity**	.90 (.01)	.90 (.01)	.87 (.01)	.58 (.05)	.74 (.02)
**pRF size**	.36 (.02)	.37 (.03)	.49 (.03)	.49 (.03)	.38 (.02)
**CMF**	.61 (.02)	.53 (.02)	.55 (.03)	.50 (.04)	.46 (.03)
