# Supplementary material for: Intersession reliability of population receptive field estimates
Source: Neuroimage. 2016 Dec;143:293–303. doi: 10.1016/j.neuroimage.2016.09.013 (PMC5139984; doi:10.1016/j.neuroimage.2016.09.013)
Supplement: Supplementary file 1 — Supplementary material [file mmc1.docx]

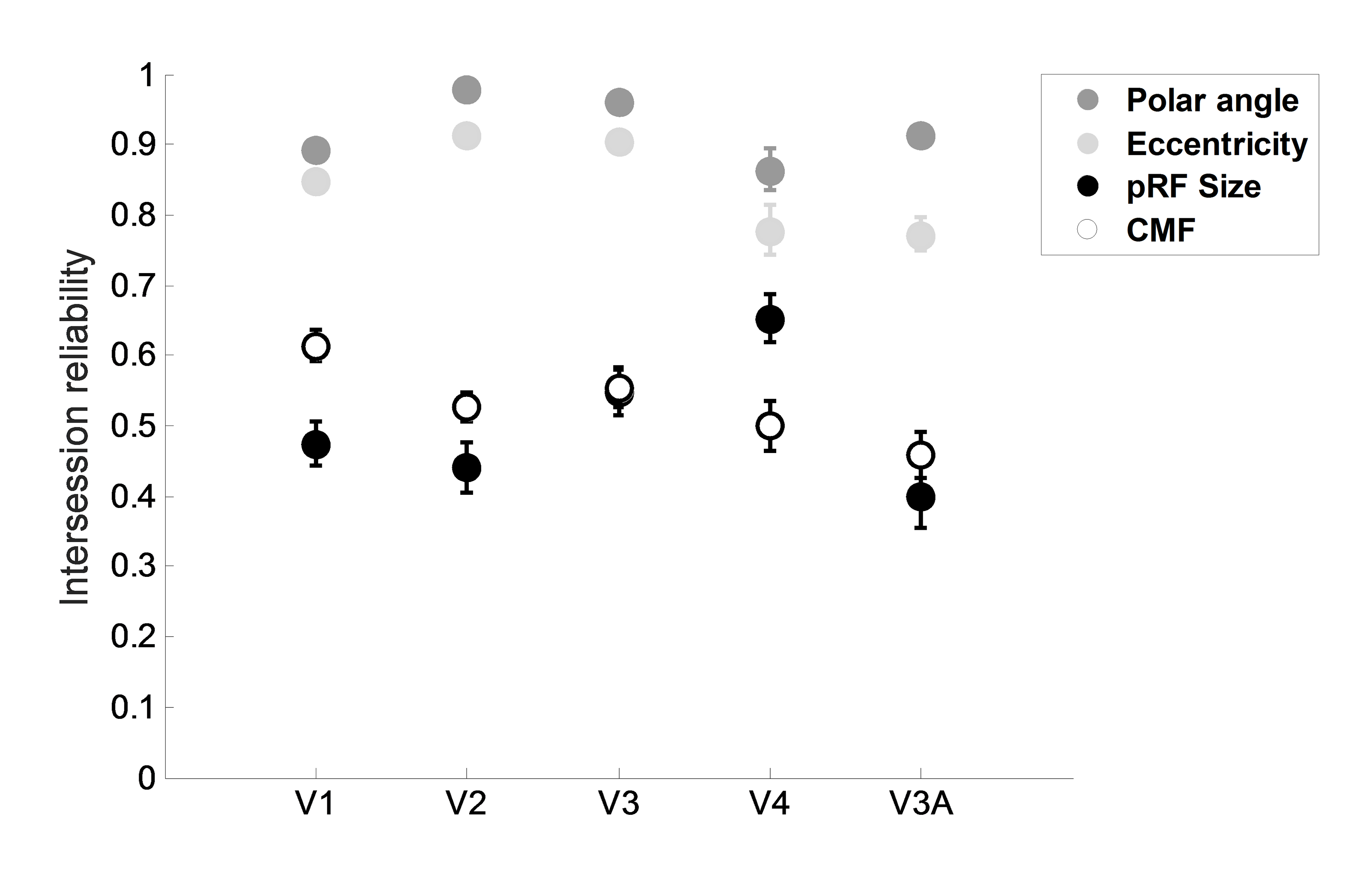


Figure S1 Intersession reliability estimates for polar angle, eccentricity, and pRF size for all regions of interest when using a canonical HRF and maps were smoothed after the pRF model fit. Error bars denote +/- 1 standard error of the mean. If there are no error bars visible, they are smaller than the symbol. For eccentricity, and pRF size, mean Spearman’s rho is displayed, while the mean circular correlation is displayed for polar angle.


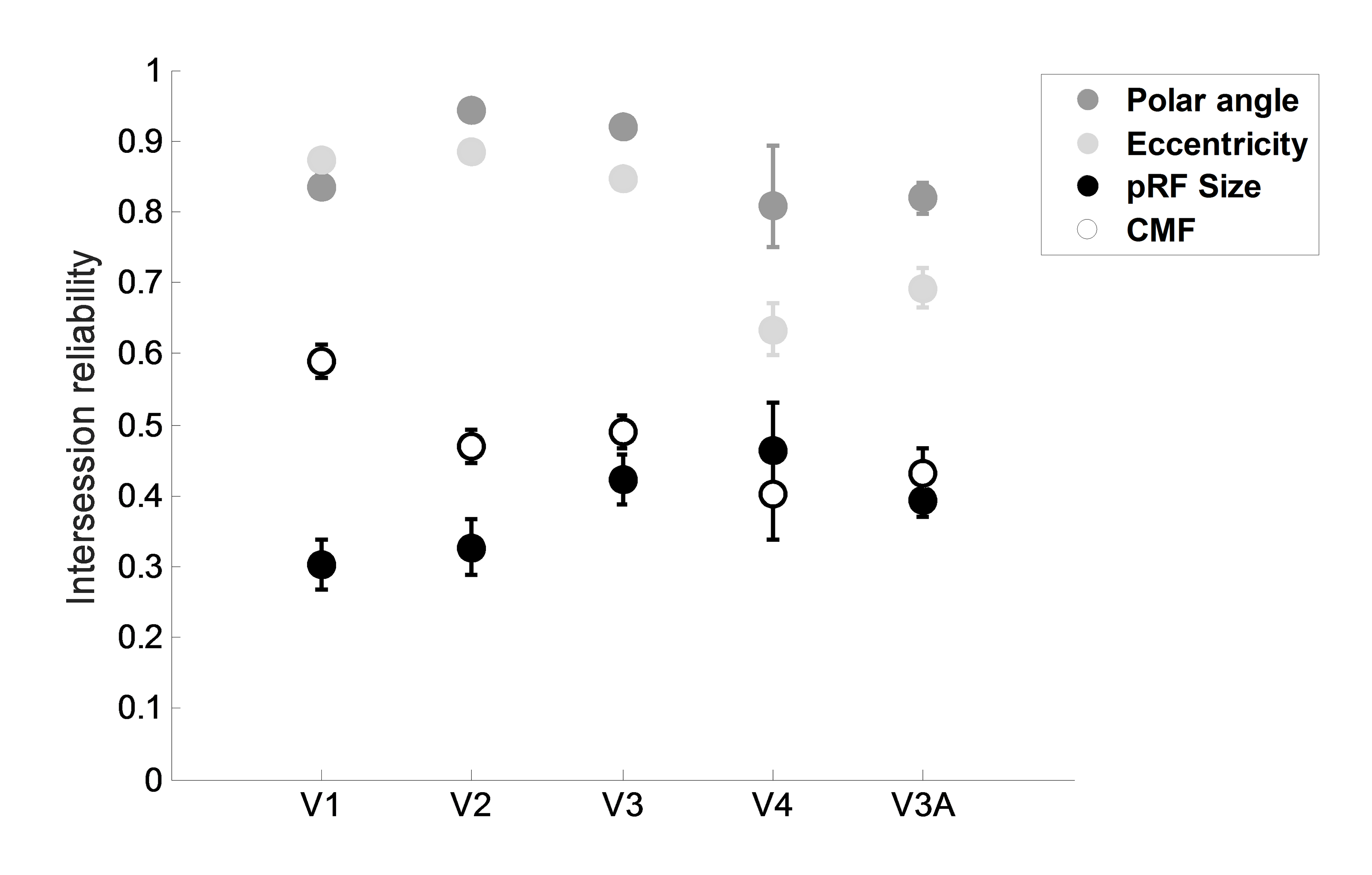


Figure S2. Intersession reliability estimates for polar angle, eccentricity, and pRF size for all regions of interest when using individual HRF for each hemisphere and session (unsmoothed data). We used a canonical HRF for the data of three (out of 64) hemispheres because it was impossible to reliably estimate an individual HRF based on the respective HRF runs. As in the main analysis parameter were spatially smoothed after the pRF model fit, Error bars denote +/- 1 standard error of the mean. If there are no error bars visible, they are smaller than the symbol. For eccentricity, and pRF size, mean Spearman’s rho is displayed, while the mean circular correlation is displayed for polar angle.


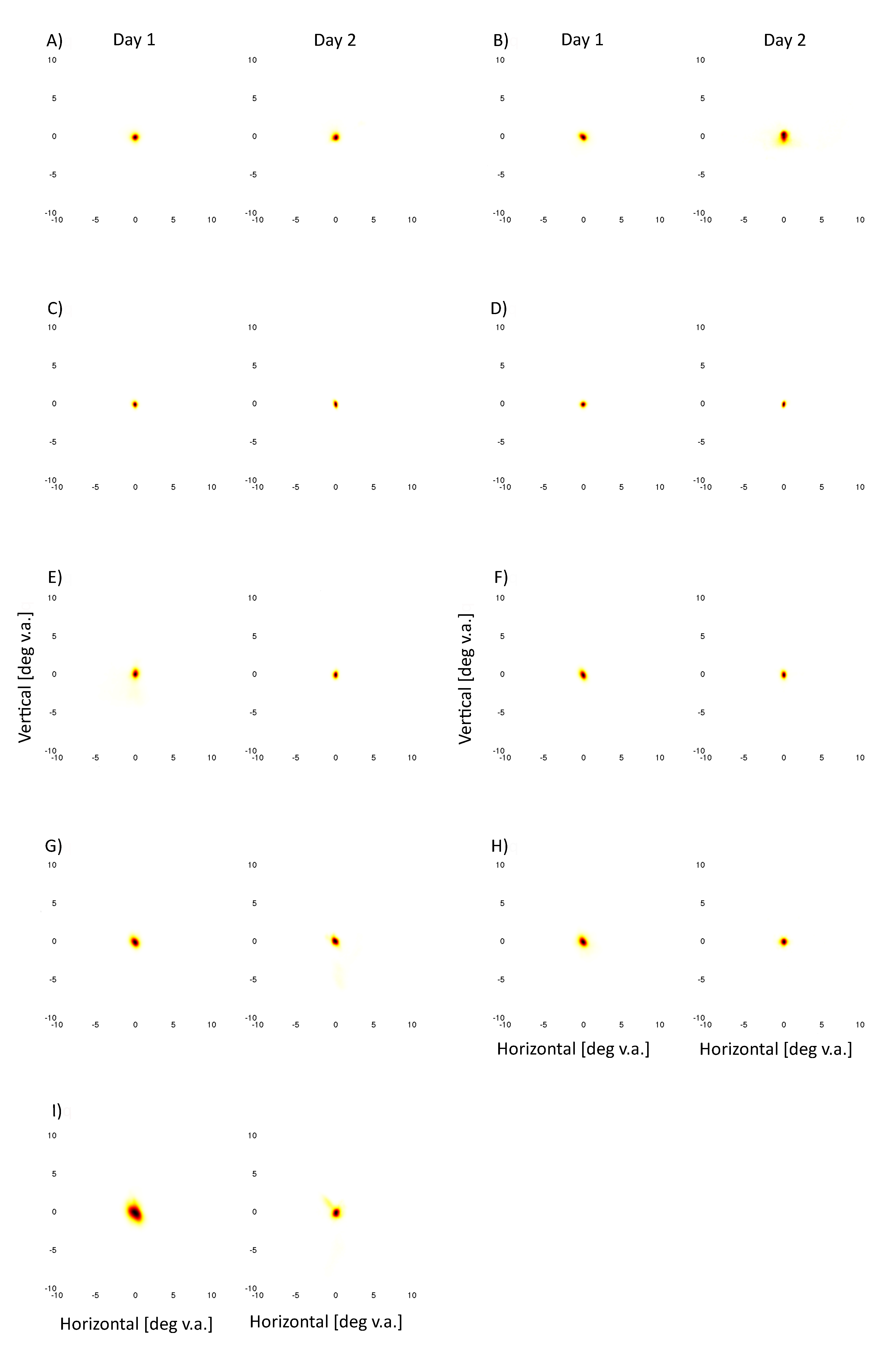


Figure S3 A)-I) center of gaze for all participants for which eye tracking data were collected during both sessions. Deviation from the fixation dot is plotted. Shading indicates the incidence of gaze on that location. Black indicates high frequency, yellow indicates low frequency. Horizontal/Vertical deg v.a = Horizontal/Vertical degree visual angle deviation from desired fixation. .


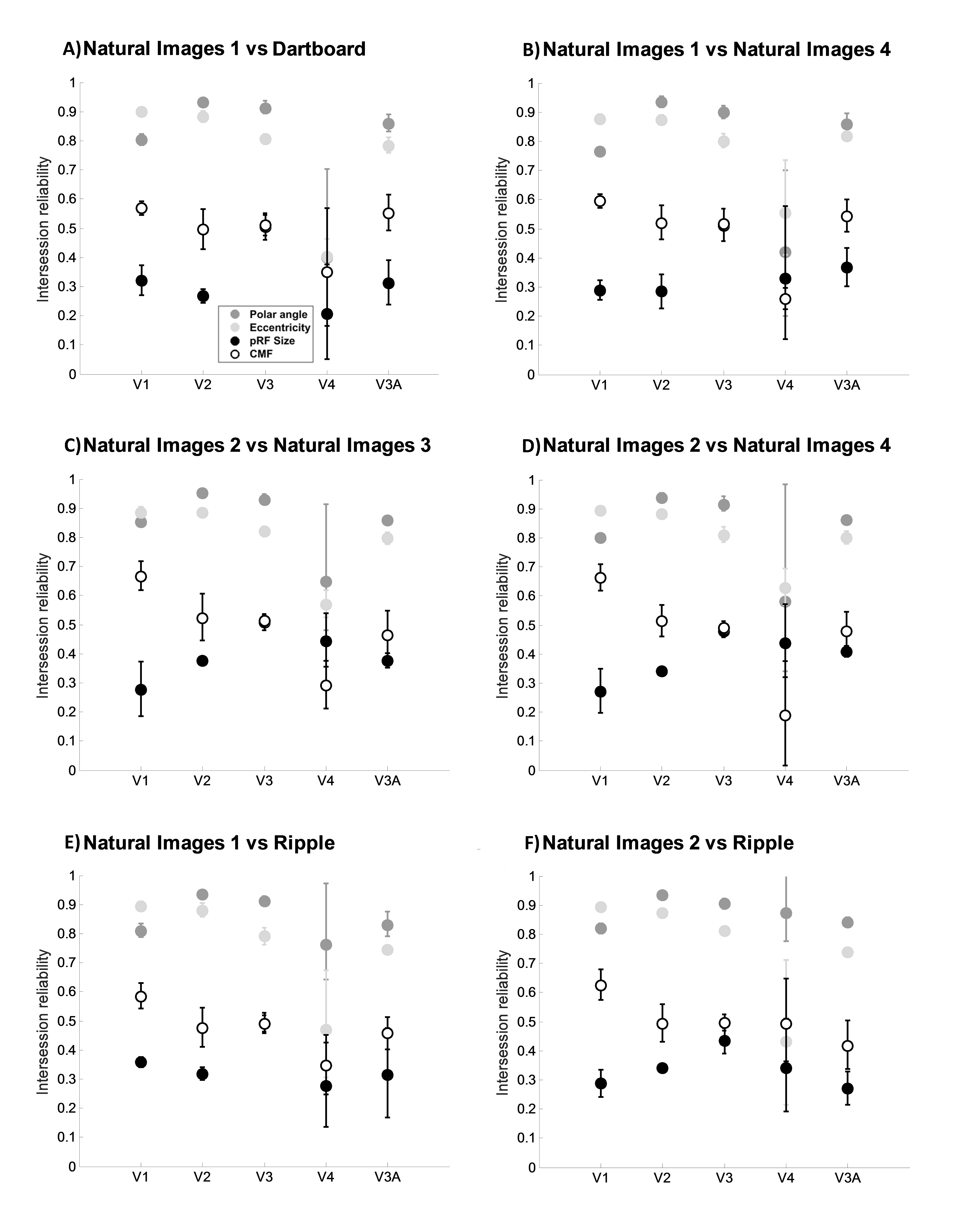

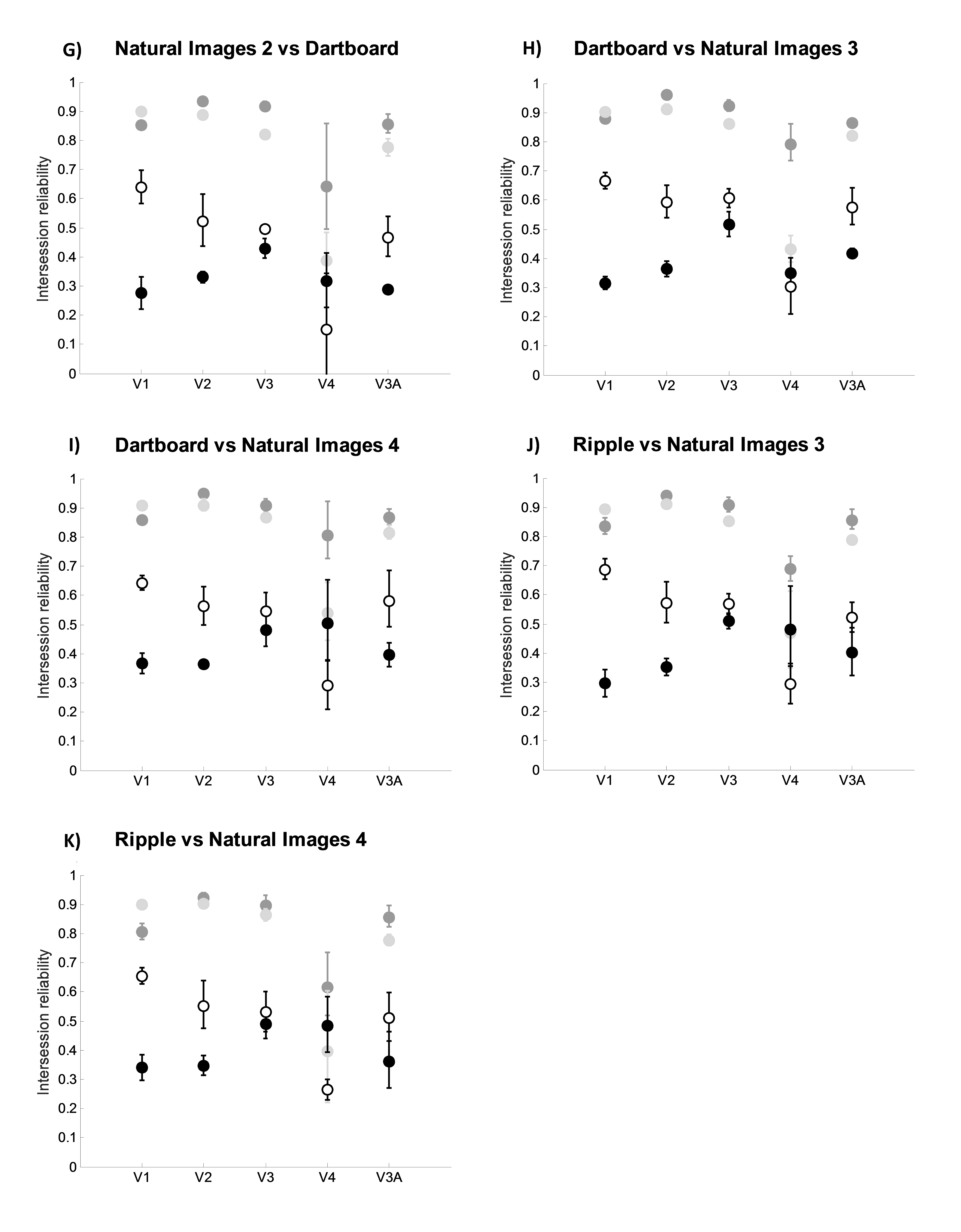


*Figure S4: Intersession reliability estimates comparing different mapping stimuli/sessions (n=6 hemispheres). Error bars denote +/- 1 standard error of the mean. If there are no error bars visible, they are smaller than the symbol. Symbols denote average intersession reliability for polar angle (dark gray), eccentricity (light gray), pRF size (black) and CMF (white) estimates. For eccentricity, pRF size, and CMF, mean Spearman’s rho is displayed, while the mean circular correlation is displayed for polar angle. Comparison of: A) first initial and dartboard carrier, B) first initial and fourth natural image session, C) second initial and third natural image session, D) second initial and fourth natural image session, E) first initial natural image session and ‘ripple’ carrier, F) second initial natural image session and ‘ripple’ carrier, G) second initial natural image session and dartboard carrier, H) dartboard carrier and third natural image session, I) dartboard carrier and fourth natural image session, J) ‘ripple’ carrier and third natural image session, K) ‘ripple’ carrier and fourth natural image session.*


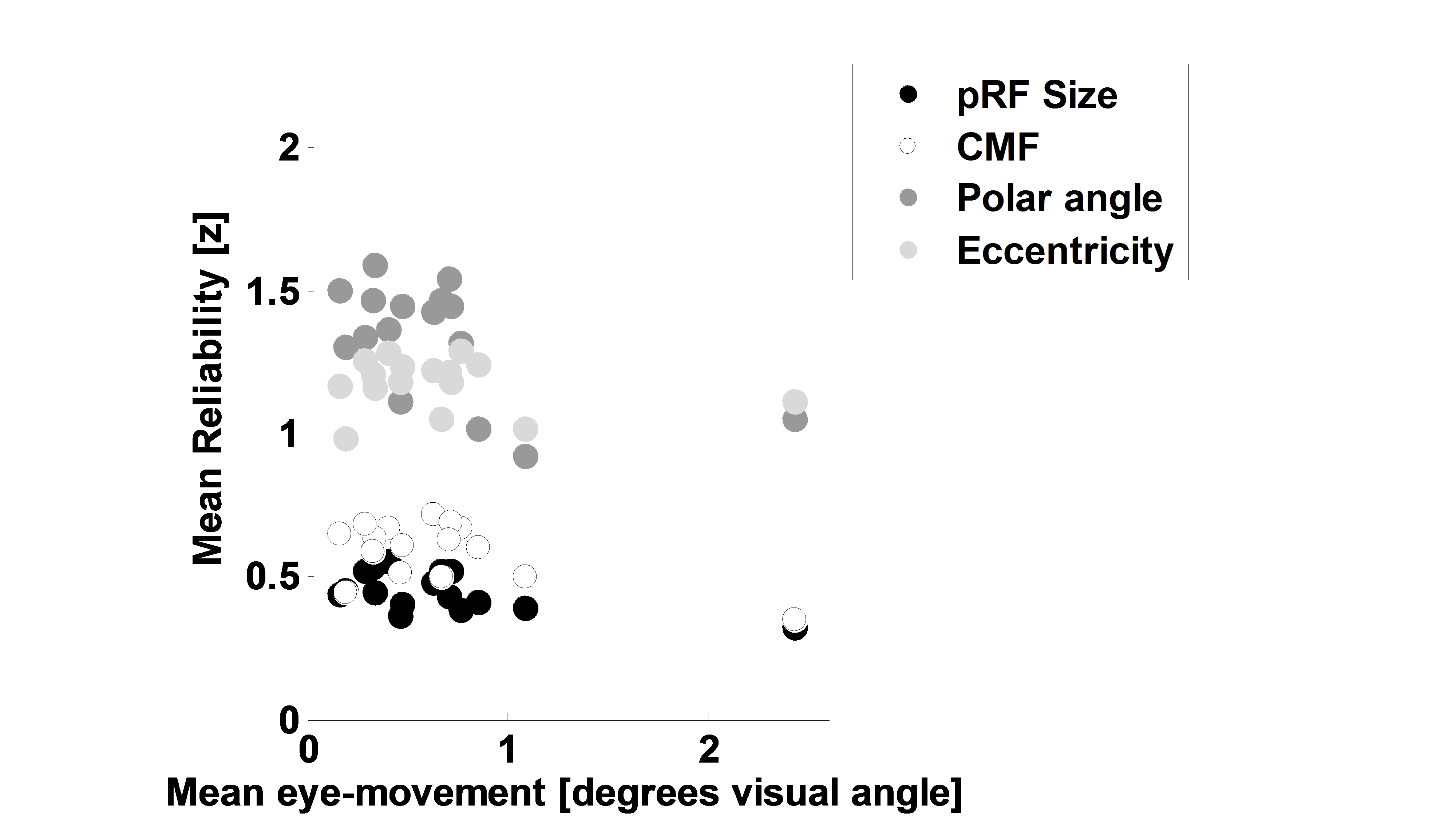


Figure S 5: Average median absolute deviation (MAD) of gaze for each participant, plotted against the mean intersession reliability estimates for pRF size, CMF, polar angle, and eccentricity. Average intersession reliability is collapsed across regions of interest.


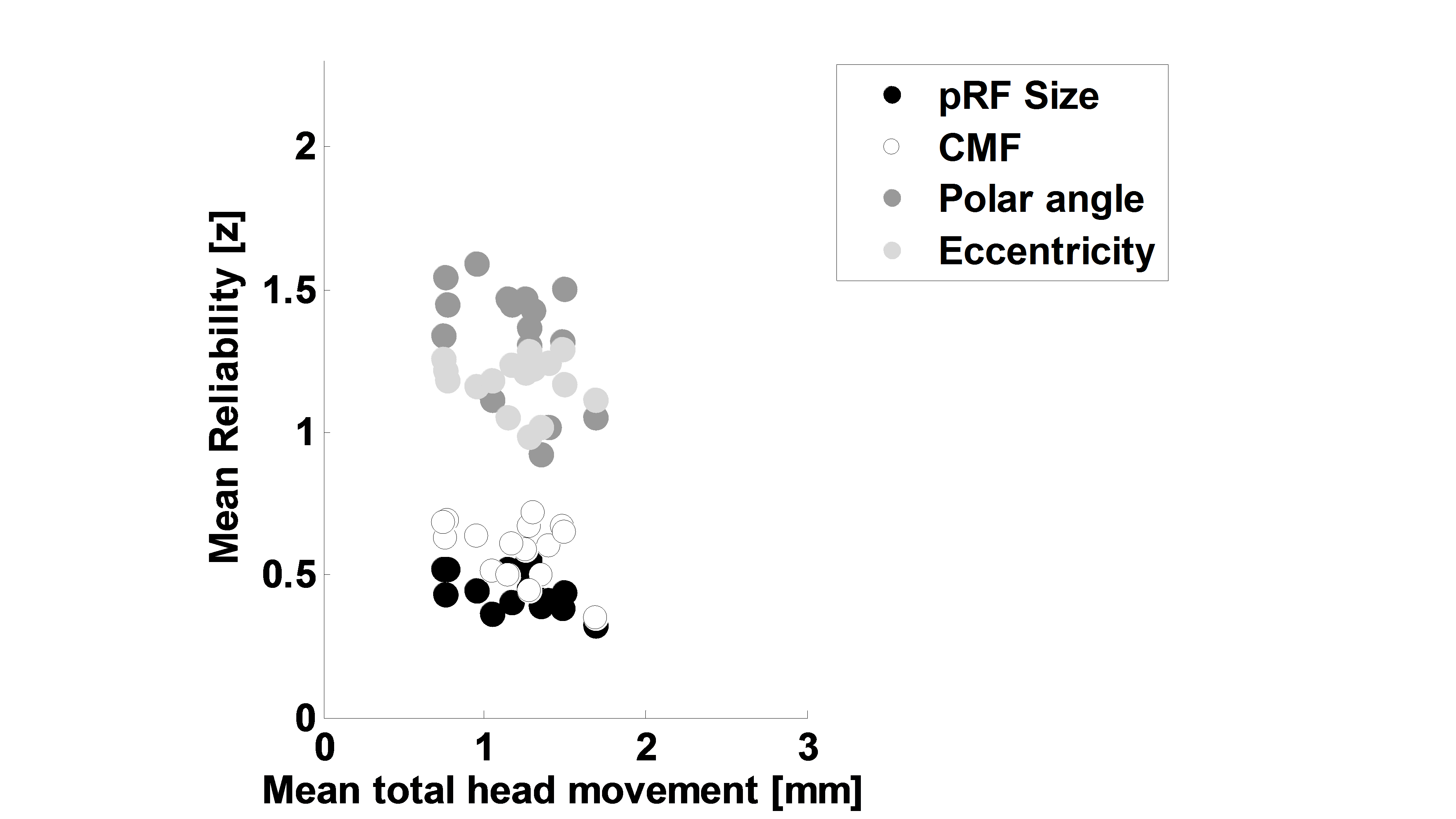


Figure S 6 Average estimated translational head motion across runs for each participant, plotted against the mean intersession reliability estimates for pRF size, CMF, polar angle, and eccentricity. Average intersession reliability is collapsed across regions of interest.


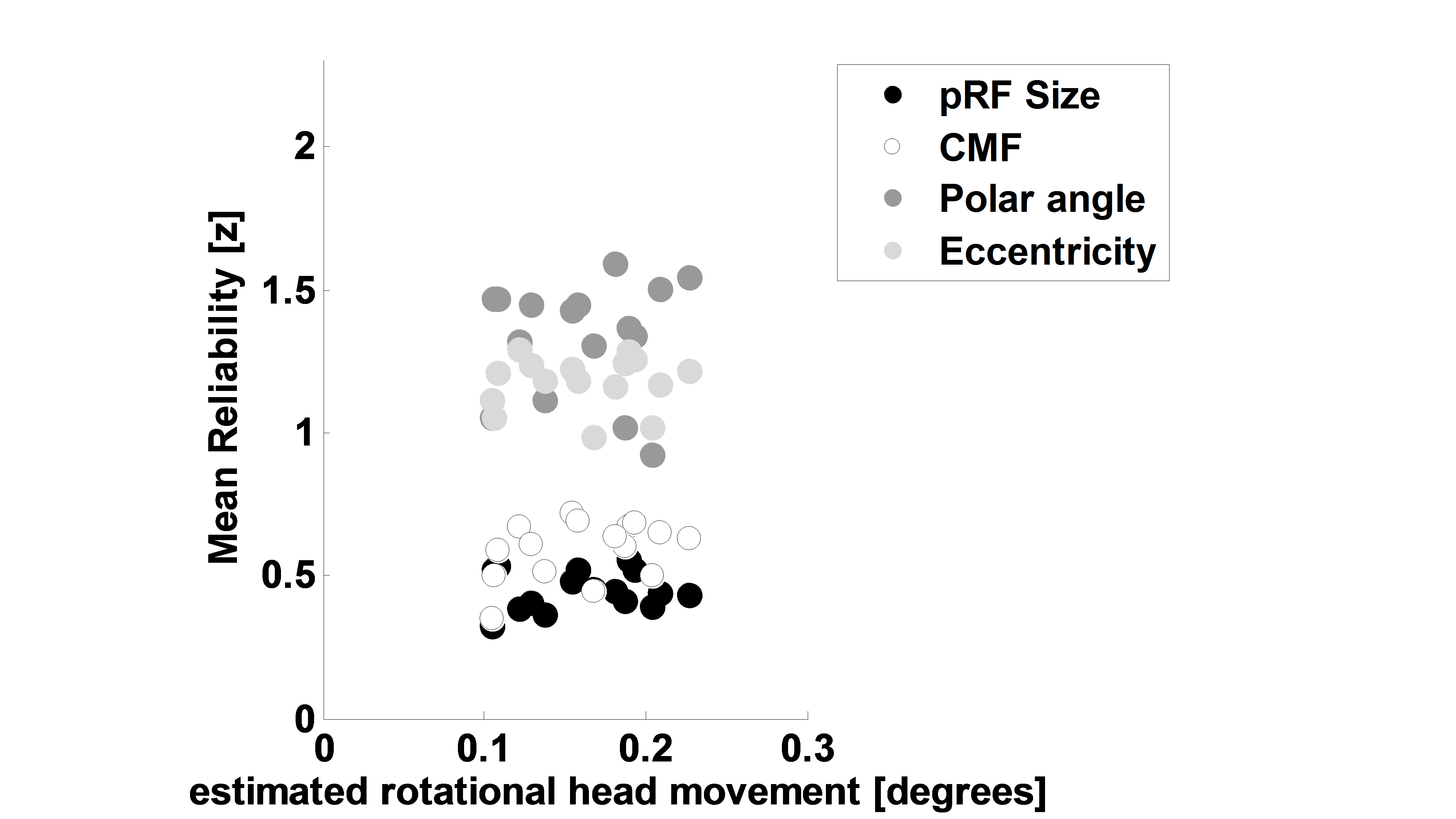


Figure S 7 Average estimated rotational head motion across runs for each participant, plotted against the mean intersession reliability estimates for pRF size, CMF, polar angle, and eccentricity. Average intersession reliability is collapsed across regions of interest.
